# Modulatory Effect of Myokines on Reactive Oxygen Species in Ischemia/Reperfusion

**DOI:** 10.3390/ijms21249382

**Published:** 2020-12-09

**Authors:** Márton Richárd Szabó, Márton Pipicz, Tamás Csont, Csaba Csonka

**Affiliations:** 1 Metabolic Diseases and Cell Signaling (MEDICS) Research Group, Department of Biochemistry, University of Szeged, Dóm tér 9, 6720 Szeged, Hungary; szabo.marton@med.u-szeged.hu (M.R.S.); pipicz.marton@med.u-szeged.hu (M.P.); csont.tamas@med.u-szeged.hu (T.C.); 2 Interdisciplinary Centre of Excellence, University of Szeged, Dugonics tér 13, 6720 Szeged, Hungary; 3 Department of Sports Medicine, University of Szeged, Tisza Lajos krt 107, 6725 Szeged, Hungary

**Keywords:** cardioprotection, brain, myocardial, ROS, conditioning, remote, exercise, FGF, IGF, LIF

## Abstract

There is a growing body of evidence showing the importance of physical activity against acute ischemic events in various organs. Ischemia/reperfusion injury (I/R) is characterized by tissue damage as a result of restriction and subsequent restoration of blood supply to an organ. Oxidative stress due to increased reactive oxygen species formation and/or insufficient antioxidant defense is considered to play an important role in I/R. Physical activity not only decreases the general risk factors for ischemia but also confers direct anti-ischemic protection via myokine production. Myokines are skeletal muscle-derived cytokines, representing multifunctional communication channels between the contracting skeletal muscle and other organs through an endocrine manner. In this review, we discuss the most prominent members of the myokines (i.e., brain-derived neurotrophic factor (BDNF), cathepsin B, decorin, fibroblast growth factors-2 and -21, follistatin, follistatin-like, insulin-like growth factor-1; interleukin-6, interleukin-7, interleukin-15, irisin, leukemia inhibitory factor, meteorin-like, myonectin, musclin, myostatin, and osteoglycin) with a particular interest in their potential influence on reactive oxygen and nitrogen species formation or antioxidant capacity. A better understanding of the mechanism of action of myokines and particularly their participation in the regulation of oxidative stress may widen their possible therapeutic use and, thereby, may support the fight against I/R.

## 1. Introduction

Tissue damage caused by ischemia/reperfusion (I/R) injury is presented in various clinical manifestations (e.g., myocardial infarction, stroke, peripheral arterial disease, ischemic nephropathy, etc.) and is considered to be a leading cause of death all over the world. Therefore, investigation of the potential anti-ischemic mechanisms, compounds, and strategies to attenuate I/R injury are extensively studied due to their great clinical importance [1,2].

I/R injury is characterized by tissue damage as a result of restriction and subsequent restoration of blood supply to an organ. During ischemia, oxygen and nutrient deprivation leads to time-dependent cell death [2]. The early restoration of blood flow salvages ischemic tissue and prevents complete damage; nevertheless, the reperfusion induces additional cell death to the ischemic organ damage [2]. Accumulation of reactive oxygen species (ROS) and the consequent oxidative stress prominently contribute to the cell death in I/R injury.

### 1.1. ROS in I/R Injury

ROS are oxygen-containing reactive molecules generated in biological systems mostly by reduction in molecular oxygen [3]. The most important representatives of ROS are superoxide anion, hydrogen peroxide (H_2_O_2_), and hydroxyl radical. Reactive nitrogen species (RNS) contain nitrogen, and these molecules are usually derivatives of nitric oxide (NO) or peroxynitrite (ONOO^−^). ROS/RNS can be either free radicals (e.g., superoxide, hydroxyl radical, NO) or non-radical reactive species (e.g., H_2_O_2_, ONOO^−^). ROS/RNS can be generated by either enzymatic or non-enzymatic processes. Enzymatic ROS/RNS formation include the leakage of superoxide anion from mitochondrial electron transport chain, superoxide anion production via nicotinamide adenine dinucleotide phosphate (NADPH) oxidases (NOX), xanthine oxidase, cyclooxygenases, uncoupled NO synthase (NOS), and production of NO by NOS. Non-enzymatic formation of ROS/RNS includes the generation of hydroxyl radicals from H_2_O_2_ in the Fenton or Haber–Weiss reactions or the formation of ONOO^−^ via the rapid reaction of NO and superoxide anion. Endogenous antioxidants are important ROS scavengers that are responsible for maintaining ROS/RNS at normal levels. Both small molecules, such as glutathione and urate, or enzymatic systems, including superoxide dismutase (SOD), catalase, glutathione peroxidase (GPx), glutathione reductase, and heme oxygenase (HO), are effective components of cellular antioxidant mechanisms [3,4,5].

Oxidative stress is considered to play an important role in I/R injury, based on the findings that ROS is elevated in I/R, scavenging ROS protects against I/R injury, and experimental generation of ROS causes similar tissue damage to I/R (for review see [1,2,6,7]). Tissue nitro-oxidative stress develops in I/R injury as a result of excess formation or insufficient removal of ROS and/or RNS. Oxidative burst mainly occurs in reperfusion; nevertheless, ischemia is also accompanied by ROS production [2,8]. Besides, ischemia-induced enzymatic alterations and metabolic consequences promote oxidative burst at the beginning of reperfusion. Ischemia leads to diminished electron flux through the mitochondrial respiratory chain, which, in turn, causes superoxide anion formation at complex I and III [2], and results in the accumulation of reducing equivalents [8]. During the ischemic period, the xanthine oxidase substrate hypoxanthine is raised, and hypoxia inhibitory factor-1α is induced, which activates NOX [1,2,7]. Moreover, in response to hypoxia, xanthine dehydrogenase is converted to xanthine oxidase, and the NOS cofactor tetrahydrobiopterin is oxidized to dihydrobiopterin, resulting in the uncoupling of NOS [1,2,7]. At the beginning of reperfusion, the reintroduction of oxygen enhances the ROS generation, called the oxygen paradox [2]. Upon reperfusion, the accumulated reducing metabolites serve as electron donors for ROS formation in the mitochondrial respiratory chain [8]. In the presence of oxygen, xanthine oxidase converts hypoxanthine to xanthine, accompanied by the release of superoxide anion and hydrogen peroxide as well [1,2,7]. Uncoupled NOS and activated NOX enzymes also generate superoxide anion during reoxygenation [1,2,7]. Finally, the imbalance in the pro- and antioxidant systems leads to elevated ROS/RNS that may result in oxidative stress (i.e., oxidative damage of proteins, nucleic acids, and lipids) and can trigger activation of cell death pathways [1,2,7].

Modulation of I/R-associated oxidative stress by inhibiting ROS production and/or by enhancing antioxidant capacity seems to be a promising strategy to attenuate I/R injury and induce tissue protection [1,6,9]. For instance, inhibition of xanthine oxidase by allopurinol or the NOX enzyme by diphenyliodonium may exert protection against I/R injury in different tissues [1,7]. Furthermore, several gain or loss of function studies implicated the protective role of SOD-1 (cytosolic CuZnSOD), SOD-2 (mitochondrial MnSOD), and SOD-3 (extracellular CuZnSOD) in I/R injury [1]. Although many experimental studies focusing on oxidative stress modulation are promising, controversial preclinical findings exist, and clinical translational barriers should be overcome [1,7].

### 1.2. Exercise-Induced Remote Organ Protection Against I/R

Enormous effort is devoted to developing approaches limiting oxidative stress and I/R-related morbidity and mortality [9,10]. The cardiovascular beneficial effect of physical activity is well known [11,12,13]. Besides the alteration of general cardiovascular risk factors (e.g., high blood pressure or hypercholesterolemia), exercise confers direct protection against I/R injury of a distant organ. This protective effect may include the development of collateral arteries, alterations in circulation, expression of endoplasmic reticulum stress proteins, and the modulation of cyclooxygenase-2 activity, heat shock proteins, and ATP-sensitive potassium channels [14,15]. Exercise also induces antioxidant effects [16,17,18] and diminishes the increased susceptibility of cardiac mitochondria to undergo permeability transition pore opening [19]. These protective phenomena collectively are also called exercise-induced conditioning [20]. In skeletal muscle, the active use of the contractile apparatus, i.e., physical work or exercise training, leads to the release of a high amount of different skeletal muscle-derived humoral factors (e.g., myokines, metabolites, non-coding regulatory RNAs, exosomes, etc.) to regulate a variety of metabolic and cellular processes in various tissues and organs of the body, including the adipose tissue, bone, brain (central nervous system), pancreas, liver, gastrointestinal system, heart, and even the skeletal muscle itself. The collective term ‘myokine’ was established by Pedersen in 2003 to be used for cytokines, which are produced and released by contracting skeletal muscles, exerting their effects in other organs of the body [21,22] through endocrine signaling pathways. The majority of myokines were also shown to exert protection against ischemia [20]. Moreover, the release of several muscle-derived myokines has been observed in settings of remote ischemic preconditioning, further emphasizing the promising beneficial role of myokines in I/R injury [23,24,25,26,27].

In this article, we aimed to review the impact of a set of relevant myokines [28,29] on the modulation of ROS formation in the context of I/R in various organs. We believe that providing a recent update and a systematic discussion of this topic may contribute to a better understanding of this field and may potentiate further research focusing on the possible use of myokines against I/R injury [23]. 

## 2. Modulatory Role of Myokines on ROS in the Context of I/R

We have performed a preliminary search using the PubMed database, and the results are presented in Table 1. First, we applied the name of certain myokines alone (column 2) as a search term or extended the search with the addition of the search term ‘myokine’ (column 3). Then, myokines (column 1) and ‘ischemia’ were searched (column 4) to check the possible involvement of the myokines in this field. Finally, we extended this latter search with the addition of the expressions “ROS”, “ROI”, “oxidative”, “antioxidative”, “reactive”, “nitric”, and “stress”, respectively (columns 5–11). Based on these search results, the myokines could be classified into three categories: (i) no or very weak documented associations between myokines and ischemia (for instance, in case of meteorin-like, musclin, or osteoglycin, etc.); (ii) myokines showing a marked association with ischemia and ROS (e.g., fibroblast growth factor-21 (FGF-21), irisin, leukemia inhibitory factor (LIF), etc.), and (iii) myokines with an overwhelming number of search counts, e.g., brain-derived neurotrophic factor (BDNF), fibroblast growth factor-2 (FGF-2), insulin-like growth factor-1 (IGF-1), and interleukin-6 (IL-6). However, this latter group may show deceptive values since these myokines were originally identified as signaling molecules released by non-muscular cells; for instance, IL-6 is also a well-known pro-inflammatory cytokine secreted independently from the skeletal muscle. Therefore, the interpretation of their ischemia/ROS connection needs further careful analysis. In the following paragraphs, we introduce the effects of the different myokines and the current position on their possible relation to the oxidative stress modulation in the context of I/R.

### 2.1. Interleukin (IL) 6

IL-6 is a multifunctional cytokine released into the blood circulation in response to physical activity, and it is considered to be the first myokine with pleiotropic effects on different tissues [30]. IL-6 binds to the IL-6 receptor (IL-6R) and gp130 receptor complex and activates diverse signaling pathways, including JAK/STAT3 pathway [30]. STAT3 transcription factor seems to be involved in cardioprotection and upregulates antioxidants (e.g., SOD-2, metallothionein) [31,32]. In connection, the antioxidant property of IL-6 has been proposed in some studies as well [33,34,35].

IL-6 has been found as a mediator of exercise-induced cardioprotection against myocardial I/R injury (Table 2) [36]. In line with these observations, few studies showed that IL-6 administration indeed induces cardioprotection [37,38]. An IL-6 pretreatment of 10 ng/mL protected cardiomyocytes against simulated I/R in a NO-dependent manner since the selective inhibition of NOS-2 (iNOS) abolished the protection [38]. IL-6 pretreatment increased NOS-2 expression and did not alter the NOS-1 (nNOS) and NOS-3 (eNOS) isoforms [38]. Mitochondrial function, which correlates to ROS formation, was also improved by IL-6 in a NO-dependent manner [38]. A loss-of-function study demonstrated that IL-6 has a pivotal role in ischemic preconditioning via JAK-STAT signaling and upregulation of NOS-2 and cyclooxygenase-2 (COX-2) [39]. Interestingly, McGinnis et al. showed that IL-6-mediated exercise-induced cardioprotection did not change myocardial NOS-2 or cyclooxygenase-2, and they suggested other possible downstream mechanisms [36]. 

It has been demonstrated that IL-6 is neuroprotective in cerebral ischemia [40,41,42,43], so it is plausible that exercise-induced IL-6 release contributes to the protection against ischemic stroke. In vivo administration of 50 ng IL-6 reduced cerebral infarction, restored SOD-2 expression via IL-6R-STAT3 pathway, and blocked the oxidation and dissociation of IL-6R and gp130 [40]. They suggested that the oxidation and subsequent disruption of the IL-6 receptor contribute to I/R injury and IL-6 protects against oxidative stress by preserving IL-6 receptor complex integrity and by the upregulation of antioxidants (e.g., SOD-2) [40]. Similar findings were obtained in oxygen–glucose deprivation (OGD)-primary neuronal cells treated with IL-6 [40]. In neuronal stem cells, IL-6 increased the activation of STAT3, expression of SOD-2, and decreased superoxide anion production [44]. These mechanisms were in casual relation to the cytoprotection against OGD and in vivo ischemic stroke [44]. 

IL-6 also seemed to participate in the protection against I/R injury in the liver and be associated with ROS modulation [45,46,47]. IL-6 treatment decreased oxidative stress–responsive HO-1 and NF-kappaB, accumulation of MDA, and increased GSH content in fatty liver subjected to I/R [47]. MnSOD (SOD-2) was increased in response to IL-6 in hypoxia/reoxygenation-exposed hepatocytes via STAT3 pathways [46]. Interestingly, Tiberio et al. suggested that Hsp70 expression may play an important role in the IL-6-induced hepatoprotection [45], partly because Hsp70 increases antioxidant GSH content by the modulation of glutathione-related enzymes [48].

### 2.2. Irisin

Irisin, the derivative of the fibronectin type III domain-containing protein 5 (FNDC5), was first described in 2013 [49]. Irisin is capable of inducing changes in adipose tissue [50,51] by increasing the expression of uncoupling protein-1 (UCP-1), thereby leading to the conversion of white adipose tissue to brown adipose tissue (by a process termed browning) and resulting in the formation of the beige adipose tissue. Irisin also increases thermogenesis, metabolism of lipids and glucose, and reduces adipogenesis [52,53]. The intracellular signaling pathway of browning is based on the phosphorylation of mitogen-activated protein kinases (MAPKs), such as extracellular signal-regulated kinases (ERKs) and p38 protein [50,54].

The potential protective effect of irisin in ischemia (and reperfusion), particularly in the myocardium via ROS formation, has been presented by a few papers (Table 3). Wang and colleagues found that exogenous irisin administration (100 μg/kg intraperitoneally) plays a pivotal role in protecting the heart against I/R injury by increasing SOD-1 levels in C57/BL6 mice [55]. Similarly, another study showed that 1 μg/kg intravenous administration of exogenous irisin produced dose-dependent protection against I/R-induced injury to the heart, reduced total ROS production, and simultaneously increased SOD activity [56]. They also found that in the presence of heat-denatured irisin, the protective effects on cardiac I/R injury were lost. In another paper, irisin treatment reduced the levels of ROS and increased the levels of components of the antioxidative system, including glutathione, SOD, and GPx via the dynamin-like GTPase optic atrophy 1 protein [57]. Administration of exogenous irisin also conferred protection against I/R-induced injury and oxidative stress in the lung [58]. Irisine at 12.5–50 nmol/L concentration mitigated oxygen–glucose deprivation (OGD)-induced neuronal injury and reversed OGD-induced oxidative stress in neuronal cells [59], and in a similar model, it suppressed the levels of nitrotyrosine, superoxide anion, and 4-hydroxynonenal (4-HNE) in peri-infarct brain tissues [60]. Intravenous irisin administration reduced malondialdehyde and myeloperoxidase levels but increased SOD and GPx activities in a mouse model of intestinal I/R injury [61] and significantly decreased the ROS accumulation. It decreased the levels of MDA, XO, and 4-HNT, while it increased the levels of SOD and GPx in intestinal tissues after gut I/R which was neutralized by irisin antibody [62]. Exogenous irisin significantly decreased oxidative stress in hepatic I/R [63,64,65]. Irisin given intraperitoneally at 250 μg/kg concentration reduced ER stress and oxidative stress after renal I/R, which was associated with the upregulation of GPx-4. TRSL3 (a GPx4 inhibitor) reduced the expression of GPx4 and abolished the protective effects of irisin in I/R-induced acute kidney injury, suggesting that GPx4 is a vital component in irisin’s protective effect after renal I/R. [66]. Irisin administration at a concentration of 100 μg/kg increased SOD and reduced MDA levels relative to those mice in the renal I/R group, indicating that irisin has a protective effect on the kidney by inhibiting oxidative stress in I/R injury [67].

### 2.3. Brain-Derived Neurotrophic Factor (BDNF)

It is well accepted that muscle contraction-induced myokine release, particularly BDNF, has neurotrophic and neuroprotective effects [68]. The neurotrophic factor BDNF is an important regulator for the development of brain circuits, neuronal development, synaptic and neuronal network plasticity, as well as for neuroregeneration and neuroprotection and modulation of synaptic activity [69]. Independently of the neuronal properties, neurotrophins exert unique cardiovascular activities. The heart is innervated by sensory, sympathetic, and parasympathetic neurons, which require neurotrophins during early development and in the establishment of mature properties, contributing to the maintenance of cardiovascular homeostasis. The identification of molecular mechanisms that are regulated by neurotrophins and involved in the crosstalk between cardiac sympathetic nerves, cardiomyocytes, cardiac fibroblasts, and vascular cells has a fundamental importance in normal heart function and direct the response of the cardiovascular system to acute and chronic injury [70,71]. It is well accepted that BDNF acts on its downstream target receptor, the tropomyosin receptor kinase B, thereby activating the (BDNF)/TrkB pathway.

Based on a Pubmed search, a high number of hits were found for the term ‘BDNF’ and ‘ischemia’ (1092). Therefore, we expected a lot of new or possible links between BDNF and ROS. However, only a few articles were found showing direct evidence between BDNF and ROS-induced regulation of ischemia (Table 4). González-Rodríguez and Ugidos investigated exogenous BDNF administration in ex vivo rat brain slices subjected to oxygen and glucose deprivation (OGD) models. They showed that BDNF in a concentration of 50 ng/mL given into the medium was capable of conferring neuroprotective effect and preventing oxidative stress characterized by decreased ROS production (tissue ROS production, NADPH oxidase activity, and lipid peroxidation) [72]. The direct effect of BDNF on ROS was also shown in PC12h cells. BDNF did not seem to change the total amount of ROS in the cells treated with xanthine and xanthine oxidase but increased superoxide anion and decreased H_2_O_2_ levels in neurons of the central nervous system, suggesting that reducing the conversion from superoxide anion to H_2_O_2_ is also critical for the protection by BDNF [73]. In contrast to these findings, others found that the expression and activation of NADPH oxidase were increased after a 2-day exposure to BDNF. In primary cortical cell cultures, BDNF produced oxidative stress in cortical neurons through NADPH oxidase-mediated production of superoxide anion, and BDNF induced expression and activation of NADPH oxidase, which caused oxidative neuronal necrosis [74]. A similar finding was published in human vascular endothelium: BDNF induced NOX-derived ROS generation through activation of p47 phox in a TrkB receptor-dependent manner, which led to the promotion of angiogenic tube formation possibly via Akt activation [75].

The potential protective role of BDNF against ischemia was proved by the BDNF mimetic 7,8-dihydroxyflavone (7,8-DHF). Intraperitoneal administration of 5 mg/kg 7,8-DHF two days after ischemia for four weeks attenuated cardiac dysfunction and cardiomyocyte abnormality of myocardial ischemic mice. Moreover, 7,8-DHF reduced cell death, accompanied by inhibiting mitochondrial superoxide anion generation [76]. Furthermore, exercise-induced BDNF confers certain aspects of its cardioprotective effects through the activation of the BDNF/TrkB axis in a NO-dependent manner [77].

We found a lot more studies from recent years investigating the relation between BDNF and ROS in ischemia. However, these studies are less relevant, because no causative relation was demonstrated between BDNF and ROS. In all of these papers, the central nervous system or the neurons represented the endpoints of the studies, and exclusively rodent animal models (rats and mice) were used with a wide range of secondary end-points, i.e., oxidative stress [78,79,80], SOD [81,82,83], NOS [84,85], HO-1 [86,87], or others [88,89,90].

### 2.4. Follistatin-Like 1

Follistatin-like 1 (FSTL-1) is a TGF-β1 induced, secreted glycoprotein that belongs to the follistatin family of proteins [100]. FSTL-1 is expressed and secreted by human skeletal muscle cells after physical activity. Serum FSTL-1 level is increased after 60 min of cycling [101] and high-intensity all-out sprint interval training [102], while 11 weeks of strength training enhanced FSTL-1 mRNA expression in *vastus lateralis* and *trapezius* muscles [103]. Several articles reported the possible involvement of FSTL-1 against ischemic diseases [104,105,106]. Resistance exercise-stimulated skeletal muscle-derived FSTL-1 not only reached the heart via the circulation but also reverted post-AMI cardiac remodeling and improved cardiac angiogenesis [107,108]. Both intravenous administration and transgenic overexpression of FSTL-1 protein protected cardiomyocytes against I/R injury through the Akt/AMPK pathway with subsequent NOS-3 activation and suppressed apoptosis and inflammatory response [109,110]. However, some studies refer to FSTL-1 as a cardiokine rather than myokine, appointing the heart as the major source of circulating FSTL-1 protein since its secretion is increasing rapidly after myocardial injury [111,112]. Correspondingly, FSTL-1 derived from mesenchymal stem cells protected against I/R in vivo and in vitro, enhanced SOD activity, and decreased malondialdehyde concentration (Table 4) [92]. Furthermore, cardiac overexpression of FSTL-1 reduced the severity of doxorubicin-induced cardiocytotoxicity via restoring the decreased protein level of nuclear factor erythroid 2–related factor-2 (NRF-2), upregulating SOD-1 and SOD-2 mRNA expression and reducing myocardial ROS, malondialdehyde, and 4-hydroxynonenal levels [113]. Additionally, it was reported that circulating FSTL-1 in healthy individuals is positively correlated with derivatives of reactive oxidative metabolites [114], suggesting that FSTL-1 could be induced by oxidative stress. Regarding skeletal muscle, FSTL-1 promotes endothelial cell function and improves revascularization in ischemic hindlimbs through activation of Akt-NOS-3 signaling (Table 4) [91]. Moreover, treatment of C2C12 murine skeletal muscle cell line with inflammatory cytokines, such as interferon-γ and IL-1β enhanced FSTL-1 secretion [101].

### 2.5. Fibroblast Growth Factor-21

Fibroblast growth factor-21 (FGF-21) is a regulator of glucose and lipid metabolism [115] and expressed mainly in the liver [116]. Apart from its pivotal role in energy balance, FGF-21 is also synthesized [117] and even secreted by skeletal muscle after a single boost [118] and two weeks of treadmill exercise [119]. A series of studies suggested that FGF-21 exhibits diverse beneficial functions against myocardial infarction and adverse cardiac remodeling, while elevated circulating FGF-21 is also suggested to be a sensitive biomarker for the detection of I/R injury during liver transplantation [120], implying that elevation of blood FGF-21 might be considered as an endogenous adaptive response against cellular damage. Treatment of cultured cardiomyocytes and adult mice with the pro-inflammatory agent lipopolysaccharide enhanced cardiac FGF-21 protein levels resulting in upregulated expression of antioxidant genes including SOD-2 and UCP-3, as well as decreased the extent of ROS formation possibly via an autocrine manner [121]. On the other hand, FGF-21 also confers cardioprotection via an endocrine manner. Skeletal muscle-derived FGF-21 improved cardiac function reduced apoptosis and proinflammatory cytokines after myocardial infarction [122]. Furthermore, serum FGF-21 concentration is elevated after myocardial infarction. However, the major source of circulating FGF-21 is considered to be the liver and the adipose tissue [123,124]. The action of Sirtuin 1 deacetylase, which is responsible for antioxidant gene expression in cardiac tissue [125,126,127], is partially mediated by FGF-21 [121]. In line with that, in response to doxorubicin-induced oxidative stress, FGF-21 pretreatment increased the amount of cardiac Sirtuin-1, thereby preventing subsequent cellular injury [128]. The same study also revealed that pretreatment with FGF-21 suppressed chronic doxorubicin-induced 3-nitrotyrosine, 4-hydroxy-2-nonenal, and MDA levels in murine hearts. Intraperitoneal administration of recombinant human FGF-21 for 28 days enhanced both mRNA and protein levels of SOD-2 and catalase, moreover mitigated angiotensin II-induced ROS release in the murine heart [129]. Several studies implied that NRF-2 activation is essential for FGF-21 mediated antioxidant response. NRF-2 induced hepatic FGF-21 expression and secretion in obese mice [130] and even protected the liver against oxidative stress [131], while FGF-21 deficiency enhanced type 1 diabetes-induced oxidative stress in the heart [132]. Furthermore, FGF-21 supplementation prevented lipid- or diabetes-induced cardiac apoptosis and lipotoxicity-induced cardiomyopathy, possibly through inhibition of Fyn-mediated export of NRF-2 from the nucleus [133,134]. In line with that, fenofibrate treatment significantly ameliorated diabetes-induced renal oxidative stress and dysfunction, and this beneficial effect is associated with increased FGF-21 expression and subsequent activation of NRF-2 [135]. Additionally, FGF-21 treatment restored doxorubicin-induced downregulation of NRF-2, as well as its target proteins, such as NADPH quinone oxidoreductase-1, catalase, and HO-1 levels [128]. Moreover, in vitro treatment of H9C2 cardiac cell line with FGF-21 protected against H_2_O_2_-induced cell death and decreased superoxide anion formation after simulated I/R (Table 4) [93]. The proposed mechanisms for the observed cytoprotective effect might be the enhancement of autophagy and inhibition of angiopoietin-2 [136,137,138]. Another possible way of the suppression of oxidative stress induced damage is the modulation of endoplasmic reticulum (ER) stress. FGF-21 expression and secretion are increased in response to ER-stress while FGF-21 overexpression protected cardiomyocytes against ER stress [139]. Additionally, FGF-21 transcription is highly regulated by activating transcription factor 4 (ATF-4) and CCAAT enhancer-binding protein homologous protein (CHOP), two important regulators of redox homeostasis and ER-stress [140].

### 2.6. Decorin

The small leucine-rich proteoglycan decorin is part of the extracellular matrix and a positive regulator of muscle hypertrophy [141]. Secreted from myotubes during differentiation [142], long term, combined endurance and resistance exercise upregulated decorin mRNA in *vastus lateralis* muscles, also markedly elevated plasma decorin level immediately after a single boost of resistance training [141]. Beyond that, decorin may ameliorate I/R injury via reducing oxidative/nitrative stress [143]. Intraperitoneal administration of decorin before 60 min ischemia attenuated lipid peroxidation and enhanced SOD levels in the kidney (Table 4) [94]. Moreover, decorin treatment protected cultured rat cardiomyocytes exposed to simulated I/R-induced cell death [144]. In line with that, decorin conferred antioxidant properties against traumatic brain injury [145], as well as attenuated diabetes-induced cardiomyopathy and promoted angiogenesis, possibly through activating the IGF-1 receptor [146].

### 2.7. Myonectin

The recently identified myokine C1q and tumor necrosis factor-related protein 5, also known as myonectin, is predominantly expressed by skeletal muscle and may function as an endocrine factor [147]. Serum myonectin was increased after 8 weeks of aerobic exercise [148], while treadmill running for 4 weeks enhanced myonectin mRNA and protein content in the *soleus* muscle and elevated plasma myonectin level as well [149]. However, little is known about the potential modulatory effect of myonectin on I/R related oxidative damage. B6 vitamin supplementation upregulated myonectin and leukemia inhibitory factor, as well as NRF-2 mRNA expression in the *gastrocnemius* muscle, suggesting a potential role of myonectin in the antioxidant system [150]. Moreover, systemic administration, transgenic overexpression, and treatment of cultured myocytes with myonectin protected against ischemic injury via decreasing the rate of apoptosis and inflammation [149].

### 2.8. Insulin-Like Growth Factor-1 (IGF-1)

Insulin-like growth factor 1 (IGF-1) is synthesized mainly in the liver and acts as a growth and differentiation factor. IGF-1 is also synthesized by skeletal muscle after physical activity [151,152,153,154]. Apart from its prominent role in muscle hypertrophy and regeneration, IGF-1 exerts protection against oxidative stress-induced cellular injury. The dominant isoform of muscle IGF-1 minimized oxidative damage in senescent muscle via upregulating peroxisome proliferator-activated receptor gamma coactivator 1-alpha (PGC-1α), NRF-2, and Sirtuin-1 [155]. IGF-1 pretreatment of cultured rat cardiomyocytes mitigated hypoxia/reoxygenation-induced cell death, reduced subsequent oxidative stress, and inhibited MDA production (Table 4) [95]. IGF-1 overexpression suppressed the formation of H_2_O_2_, hydroxyl radical, and nitrotyrosine accumulation, thus protected against diabetic cardiomyopathy [156]. Likewise, attenuation of circulating IGF-1 levels, through knockout of liver-specific IGF-1, resulted in downregulated expression of NRF-2 and its downstream targets NADPH quinone oxidoreductase-1, γ-glutamylcysteine ligase, and HO-1 in the aorta [157]. Additionally, the same research group assessed that exposing cultured aorta segments from IGF-1 deficient mice to H_2_O_2_ or oxidized low-density lipoprotein exacerbated oxidative stress-mediated cellular injury. Controversially, isolated cardiomyocytes from hepatic IGF-1 deficient mice showed decreased ROS formation after treatment with the pro-oxidant paraquat [158]. Apart from its antioxidant properties, IGF-1 protects against I/R injury both in vivo and in vitro. Intravenous injection of IGF-1 30 min before left anterior descending artery ligation ameliorated myocardial infarct size and apoptosis [159]. Downregulation of IGF-1 during I/R through microRNA-320 and microRNA-489 aggravated the extent of myocardial infarction, ventricular remodeling, and apoptotic cell death [160,161], proposing an important IGF-1 mediated endogenous protection against ischemic cell death. This is further affirmed by the findings that genetic depletion of mouse mast cell protease 4—presumably responsible for endogenous IGF-1 degradation—alleviated myocardial infarct size, post-ischemic cardiac dysfunction and remodeling [162]. IGF-1 also confers cardioprotection in ex vivo perfused myocardium [163] since both supplementation of IGF-1 into the perfusion fluid or IGF-1 overexpression suppressed I/R injury [164,165]. Recently it was demonstrated that subcutaneous IGF-1 supplementation for 3 days improved cardiac function after myocardial infarction [166]. Transgenic overexpression of locally acting IGF-1 isoform (mIGF-1) mitigated paraquat-induced oxidative stress, alleviated cardiac MDA production, ROS levels, as well as 4-hydroxy-2-nonenal and MDA protein adducts [127,167]. The proposed mechanism may involve the activation of Sirtuin-1 deacetylase since overexpression of mIGF-1 isoform increased cardiac Sirtuin-1 protein level in both HL-1 cells and murine primary cardiomyocytes, while genetic depletion of Sirtuin-1 abolished the protection induced by mIGF-1 against paraquat-induced oxidative damage.

### 2.9. Leukemia Inhibitory Factor (LIF)

LIF belongs to the IL-6 cytokine superfamily, presumed as a pleiotropic cytokine with a wide range of activities, including modulation of cell proliferation and growth, bone formation, and neuronal protection. LIF mRNA expression is acutely induced after 6 h of concentric exercise in the *vastus lateralis* [168], thereby pointing to the skeletal muscle as a putative source of circulating LIF. Intravenous administration of LIF protected rabbit hearts against ex vivo I/R injury, moreover increased myocardial SOD-2 activity, decreased lipid oxidation, and protein carbonylation (Table 4) [96]. Additionally, LIF transfection improved cardiac recovery and enhanced the proliferation of cardiomyocytes following myocardial infarction [169]. LIF treatment mitigated middle cerebral artery occlusion [170] and focal cerebral ischemia-induced injury, in the latter via upregulating the antioxidant enzyme peroxiredoxin 4 mRNA in cultured oligodendrocytes and SOD-3 mRNA expression in cortical neurons (Table 4) [97,98]. LIF also protected PC-12 cells against Antimycin A-induced oxidative damage, reduced ROS level, and restored SOD activity [171]. Furthermore, LIF attenuated glucose-induced ROS production in podocytes, decreased NADPH oxidase generation while enhancing total SOD levels [172].

### 2.10. Fibroblast Growth Factor 2 (FGF-2)

FGF-2, also known as basic fibroblast growth factor, is a pleiotropic protein with important roles in angiogenesis, bone formation, cell differentiation, and migration. Despite lacking the secretion signal, the low molecular weight isoform of FGF-2 is a secreted protein [173,174]. Cultured myocytes exposed to in vitro mechanical stress or scratching responded with increased FGF-2 secretion in the surrounding media [175,176,177], proposing that FGF-2 might be an exercise-induced myokine [178]. Presumed to act via autocrine/paracrine manner, FGF-2 is well documented to protect against I/R. However, it remains controversial whether the skeletal muscle-derived secreted FGF-2 isoform is involved in cardioprotection. Cardiac-specific overexpression of FGF-2 conferred cardioprotection in the ex vivo perfused murine heart [179,180,181,182], while FGF-2 knockout failed to attenuate the extent of I/R injury [183]. Interestingly, it is proposed that the low molecular weight isoform of FGF-2 might have a prominent role in these beneficial effects on the myocardium [184,185]. Additionally, treatment with both low and high molecular weight isoforms of FGF-2 protected cultured rat cardiomyocytes against doxorubicin-mediated oxidative stress [186], possibly via preventing the doxorubicin-induced decrease in NRF-2 and enhancing the subsequent upregulation of HO-1 [187,188]. Intraperitoneal administration of FGF-2 markedly alleviated I/R-induced kidney damage (Table 4) [99,189]. The proposed mechanism of action might involve the attenuation of mitochondrial DNA damage as reflected by lowering the amounts of 3-nitrotyrosine and 8-hydroxy-2-deoxyguanosine generation of tubular cells in ischemic kidneys and inhibition of excessive ER-stress. FGF-2 is also involved in the protection against cerebral ischemia [190] and mitigated H_2_O_2_-induced oxidative damage of PC-12 cells, possibly through mediating ER-stress response [191].

### 2.11. Other Myokines

**Figure 1:**
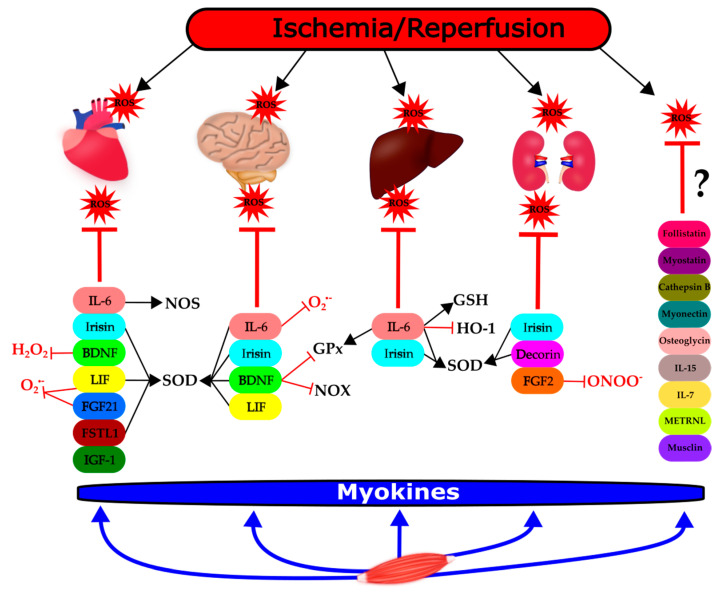
Modulation of ischemia/reperfusion-induced reactive oxygen species (ROS) formation and/or elimination by myokines in different organs. This figure shows the groups of myokines affecting ROS or antioxidant mechanisms in the heart, brain, liver, and kidneys, respectively, in the settings of ischemia/reperfusion (thick lines). The effect of the individual myokines on specific molecular targets are indicated with thin lines: T: negative effect, ↑: positive effect. BDNF: brain-derived neurotrophic factor; FGF-2: fibroblast growth factor 2; FGF-21: fibroblast growth factor 21; FSTL-1: follistatin-like; GPx: glutathione peroxidase; GSH: glutathione; HO-1: heme oxygenase-1; IGF-1: insulin-like growth factor-1; IL-6: interleukin-6; IL-7: interleukin-7; IL-15: interleukin-15; LIF: leukemia inhibitory factor; METRLN: meteorin-like; NOS: nitric oxide synthase; NOX: NADPH oxidase; ONOO^−^: peroxynitrite, O_2_^.−^: superoxide anion, SOD: superoxide dismutase, ROS: reactive oxygen species. ?: no article was found to support evidence between myokines and ROS. Figure 1 was created by the software Inkscape.

In the case of 8 other myokines, i.e., cathepsin B, follistatin, IL-7, IL-15, meteorin-like, musclin, myostatin, and osteoglycin, no relevant literature was found in the context of ROS and I/R (Table 1, Figure 1). However, their potential redox modulatory role is discussed in the next section.

## 3. Discussion

In summary, we found that several myokines confer protection against I/R injury in a variety of organs. This protection often involves the modulation of ROS/RNS formation or elimination (Figure 1).

We may conclude that some myokines exert significant effects on the redox homeostasis of tissues. Therefore, these mediators may potentially confer general protection against I/R injury. Irisin, IL-6, and BDNF are the most powerful members of these myokines. One may speculate that the use of these myokines in combination or application of physical exercise forms resulting in high irisin, IL-6, and BDNF-release would provide an even more pronounced preventive or therapeutic effect against ischemic stress. However, confirmation of these concepts needs to be done in future studies.

Importantly, all of the myokines that were investigated concerning ROS modulation were found to (i) decrease ROS formation and its harmful consequences (e.g., lipid peroxidation, etc.), or (ii) increase antioxidant enzymes protein level or activity. Superoxide anion was represented as the most frequent target of redox status according to the studies we analyzed, and among the antioxidative defense enzymes, SOD and GPx, were investigated most frequently. Taken together, the prooxidant effect of myokines could hardly be found concerning I/R (Table 2, Table 3 and Table 4).

Although in our preliminary PubMed search, we found a relatively large number of myokines in the context of I/R and ROS, it seems that only a portion of these myokines (9 out of 18) influences the redox balance significantly and are in a causative relationship with ROS/RNS formation or elimination. In several articles, the influence of myokines on ROS/RNS formation was only a secondary or tertiary finding. Moreover, in the case of several other myokines (i.e., cathepsin, follistatin, IL-7, IL-15, METRLN, musclin, myonectin, myostatin, osteoglycin), no or only very weak evidence was found to link the individual myokines to possible modulation of redox balance (Figure 1). This does not exclude the possibility that these myokines may be involved in the protection against I/R injury. However, further studies are required to clarify their precise role and mechanism of action.

In recent years, exercise training and other forms of physical activity have become one of the main clinical interventions for the prevention and treatment of (cardio)vascular diseases (sport as medicine). Therefore, a better understanding of the mechanisms underlying myokine release holds promise for the discovery of novel therapeutic targets and optimization of physical activity to improve (cardio)vascular outcomes.

## Figures and Tables

**Table 1:** Demonstration of the potential correlation between myokines and reactive oxygen species (ROS) after a Pubmed search.

Myokine		+Myokine	+Ischemia	+Ischemia +ROS	+Ischemia +ROI	+Ischemia +Oxidative	+Ischemia +Antiox	+Ischemia +Redox	+Ischemia +Nitric	+Ischemia +Stress
BDNF	25,624	77	1092	16	0	127	101	14	41	145
CTSB	6098	6	146	2	0	13	17	5	4	13
decorin	2883	23	23	0	0	0	0	0	0	2
FGF2	17,563	7	851	4	0	69	28	0	38	51
FGF21	1914	55	30	1	0	7	2	0	1	5
follistatin	2510	42	49	0	0	4	0	0	2	7
FSTL-1	386	12	18	0	0	2	0	0	1	6
IGF-1	44,896	32	716	4	0	67	34	10	28	72
IL-6	145,377	232	5070	151	2	1204	786	120	441	897
IL-7	6671	12	23	0	0	0	0	0	0	0
IL-15	6248	95	22	0	0	1	0	0	1	5
irisin	1131	466	49	5	0	18	6	3	1	18
LIF	4576	11	69	2	0	10	12	4	2	11
METRNL	66	11	2	0	0	0	0	0	0	1
myonectin	32	25	2	0	0	0	0	0	0	0
musclin	35	8	1	0	0	1	1	0	0	1
myostatin	2997	112	22	0	0	3	1	0	1	3
osteoglycin	133	2	4	0	0	0	0	0	0	0

Column 2: total number of articles obtained after searching for certain myokines, respectively. Column 3 and 4: we extended the search with the terms “myokine” and “ischemia”, respectively. Column 5–11: column 2 + the terms “ischemia” and “ROS”, “ROI”, “oxidative”, “antioxidative”, “redox”, “nitric”, or “stress”, respectively. Antiox: antioxidative; BDNF: brain-derived neurotrophic factor; CTSB: cathepsin B; FGF-2: fibroblast growth factor-2; FGF-21: fibroblast growth factor-21; FSTL-1: follistatin-like; IGF-1: insulin-like growth factor-1; IL-6: interleukin-6; IL-7: interleukin-7; IL-15: interleukin-15; LIF: leukemia inhibitory factor; METRLN: meteorin-like, ROI: reactive oxygen intermediates.

**Table 2:** Effect of interleukin-6 (IL-6) on redox state in settings of ischemia/reperfusion injury (I/R).

Species	Organ/Cell	Model	IL-6 Dose, Treatment Time, Route	Effect of IL-6 on I/R Injury	Effect of IL-6 on Redox State	Ref.
control and IL-6^−/−^ mice	heart	in vivo I/R	loss of function study	infarct size ↓, arrhythmia ↓	NOS-2 ↔, COX-2 ↔	[36]
rat	neonatal ventricular cardiomyocytes	in vitro sI/R	10 ng/mL for 6 h, 24 h before SI/R	cell death ↓	NOS-2 ↑, NOS-3 ↔, NOS-1 ↔	[38]
mice	brain	in vivo I/R	2 × 50 ng, 30 min before and 15 min after I/R, icv.	infarct size ↓	SOD-2 ↑, protein oxidation ↓	[40]
mice	primary cortical neurons	in vitro OGD	50 ng/mL, 30 min before OGD	cell death ↓	SOD-2 ↑, protein oxidation ↓	[40]
mice	neural stem cells	in vitro OGD	20 ng/mL 24 h before OGD	cell death ↓	SOD-2 ↑, O_2_^•−^↓ (DHE) *	[44]
mice	brain transplanted with neural stem cells (NSC)	in vivo I/R	NSC cells treated with 20 ng/mL 24 h before transplantation	infarct size ↓, behavior ↑	O_2_^•−^↓ (DHE) *	[44]
rat	liver	in vivo I/R	2 × 10–500 µg/kg, 12 h and 6 h before I/R, ip.	cell death ↓	HSP70 ↑, unfolded protein response ↓	[45]
rat	primary cultured hepatocytes	in vitro sI/R	10 ng/mL 24–72 h before H/R	cell death ↓	SOD-2 ↑	[46]
Zucker rat	fatty liver	in vivo I/R	500 µg/kg, 12 h or 24 h before I/R, ip.	cell death ↓	MDA ↓, GSH ↑, HO-1 ↓, NF-kB↓	[47]

COX-2: cyclooxygenase-2, DHE: Dihydroethidium assay, GSH: glutathione, HO: heme oxygenase, HSP70: heat shock protein 70, ip.: intraperitoneally, icv.: intracerebroventricularly, MDA: malondialdehyde, SOD-2: MnSOD, NF-kB: nuclear factor kappa-light-chain-enhancer of activated B cells, NOS-1, NOS-2, and NOS-3: neuronal/inducible/endothelial nitric oxide synthase, O_2_^•−^: superoxide anion, OGD: oxygen-glucose deprivation, sI/R: simulated ischemia/reperfusion, SOD: superoxide dismutase, ↑ and ↓indicate increase and decrease, respectively; ↔ indicates no changes; * indicates the detection method.

**Table 3:** Effect of Irisin on redox state in settings of I/R.

Species	Organ/Cell	Model	Irisin Dose, Treatment Time, Route	Effect of Irisin on I/R Injury	Effect of Irisin on Redox State	Ref.
mice	heart	ex vivo I/R	100 µg/kg, 30 min before I/R, ip.	infarct size ↓, function ↑	SOD-1 ↑	[55]
rat	heart	in vivo I/R	1 µg/kg immediately before reperfusion, iv.	infarct size ↓, function ↑	total SOD ↑, SOD-1 ↔, SOD-2 ↑	[56]
rat	H9c2 cardiomyoblasts	in vitro A/R	100 ng/mL, during reoxygenation	cell death ↓	ROS ↓ (DCFH-DA/DHE) *	[56]
N.A.	primary cardiomyocytes	in vitro H/R	N.A.	cell death ↓	ROS ↓ (CM-H2-DCFDA) *, GSH ↑, total SOD ↑, GPx ↑	[57]
control and UCP-2^−/−^ mice	lung	in vivo I/R	1 µg/kg immediately after ischemia, iv.	function ↑, edema ↓	ROS ↓ (DHE) *, UCP-2 ↑	[58]
human	A549 lung epithelial cell	in vitro A/R	0.1 μg/mL immediately after anoxia	apoptosis ↓	O_2_^•−^↓ (MitoSOX) *	[58]
rat	PC12 neuronal cells	in vitro OGD	12.5–50 nmol/L before OGD	cell death ↓, apoptosis ↓	ROS ↓ (ROS assay) *, MDA ↓, total SOD ↑	[59]
mice	brain	in vivo I/R	0.2 μg/g, 30 min after ischemia, iv.	infarct size ↓, inflammation ↓	O_2_^•−^ ↓ (superoxide assay) *, MDA ↔, 4-HNE ↓, nitrotyrosine ↓	[60]
mice	intestine	in vivo I/R	10ng/g or 100ng/g, 30 min before I/R, iv.	morphology ↑, inflammation ↓	MDA ↓, MPO ↓, total SOD ↑, GPx ↑	[61]
rat	IEC-6 intestinal epithelial cells	in vitro H/R	10 ng/mL 24 h before hypoxia	cell death ↓	MDA ↓, total SOD ↑, GPx ↑	[61]
mice	intestine	in vivo I/R	250 μg/kg, beginning of reperfusion, iv.	gut barrier function ↑	4-HNE ↓, MDA ↓, total SOD ↑, GPx ↑, XOR ↓	[62]
human	Caco-2 colon cells	in vitro H/R	10 nmol/L, beginning of reoxygenation	cell death ↓	ROS ↓ (DHE) *	[62]
rat	liver	in vivo I/R	250 μg/kg, beginning of reperfusion, iv.	cell death ↓, inflammation ↓	MDA ↓, total SOD ↑, GPx ↑	[63]
human	HL-7702 hepatocytes	in vitro H/R	100 ng/mL, during reoxygenation	apoptosis ↓	ROS ↓ (DHE) *, UCP-2 ↑	[63]
rat	liver	in vivo I/R	250 μg/kg, beginning of reperfusion, iv.	cell death ↓, inflammation ↓	MDA ↓, total SOD ↑, GPx ↑	[64]
mice	liver	in vivo I/R	250 μg/kg, beginning of reperfusion, ip.	cell death ↓	ROS ↓ (DHE) *, GPx ↑	[65]
mice	kidney	in vivo I/R	250 μg/kg, beginning of reperfusion, ip.	cell death ↓, function ↑, inflammation ↓	ROS ↓ (DHE) *, total SOD ↑, GPx ↑	[66]
mice	kidney	in vivo I/R	10, 100, and 200 μg/kg/day for 14 days before I/R, ip.	tubular injury ↓, function ↑	MDA ↓, UCP-2 ↑, MPO ↓, total SOD ↑	[67]

4-HNE: 4-hydroxynonenal, A/R: anoxia/reoxygenation, DHE: Dihydroethidium assay, GPx: glutathione peroxidase, H/R: hypoxia/reoxygenation, ip.: intraperitoneally, iv.: intravenously, MDA: malondialdehyde, SOD-1: CuZnSOD, SOD-2: MnSOD, MPO: myeloperoxidase, N.A.: not available, O_2_^•−^: superoxide anion, OGD: oxygen–glucose deprivation, ROS: reactive oxygen species, SOD: superoxide dismutase, UCP-2: mitochondrial uncoupling protein 2, XOR: xanthine oxidoreductase, ↑ and ↓indicate increase and decrease, respectively; ↔ indicates no changes; * indicates detection method.

**Table 4:** Effect of different myokines on redox state in settings of I/R.

Myokine	Species	Organ/Cell	Model	Myokine Dose, Treatment Time, Route	Effect of Myokine on I/R Injury	Effect of Myokine on Redox State	Ref.
BDNF	rat	brain hippocampus	ex vivo OGD	50 ng/mL after OGD	cell death ↓	ROS ↓ (CellRox) *, NOX ↓, GPx ↓, MDA+4-HNE ↓, TAC ↓, SOD-2 ↓	[72]
	rat	brain cortex	ex vivo OGD	50 ng/mL after OGD	cell death ↓	ROS ↔ (CellRox) *, NOX ↔, GPx ↔, MDA+4-HNE ↔, TAC↔, SOD-2↑	[72]
	rat	H9c2 cardiomyoblasts	in vitro H_2_O_2_^1^	100 µM^2^ 24 h before H_2_O_2_	cell death ↓	mitochondrial O_2_^•−^ ↓ (MitoSox) *	[76]
FSTL-1	mice	adductor muscle	hindlimb ischemia	skeletal muscle-derived transgenic FSTL-1	endothelial cell function ↑, revascularization ↑	NOS-3 ↑	[91]
	rat	heart	in vivo I/R	1 × 10^6^ mesenchymal stem cells injected into the abdomen under I/R	infarct size ↓, apoptosis ↓	MDA ↓, total SOD ↑	[92]
FGF-21	rat	H9C2 cardiomyoblasts	in vitro H/R	0.25, 1 or 4 μg/mL during 6 h reoxygenation	cell death ↓, apoptosis ↓	O_2_^•−^↓ (DHE) *	[93]
Decorin	rat	kidney	in vivo I/R	100 μg/kg for 9 days after reperfusion, ip.	TGF-β1 ↓, apoptosis ↓	lipid peroxidation ↓, total SOD ↑	[94]
IGF-1	rat	primary cardiomyocytes	in vitro H/R	1 h pretreatment with 100 nM	mitochondrial function ↑	MDA ↓	[95]
LIF	rabbit	heart	ex vivo I/R	5 × 10^7^ U/mg, 24 h prior to heart isolation, iv.	tension recovery ↑	protein carbonylation ↓, TBARS ↓, SOD-2 ↑	[96]
	rat	cultured oligodendrocytes	in vitro OGD	cotreatment with 200 ng/mL LIF during 24 h OGD	LDH release ↓	Prdx4 ↑, total SOD ↓	[97]
	rat	brain	in vivo I/R	125 μg/kg at 6, 24, and 48 h after I/R, iv.	N.A.	SOD-3 ↑, total SOD ↑	[98]
	rat	primary cortical neurons	in vitro OGD	pretreatment with 200 ng/mL	LDH release ↓	SOD-3 ↑	[98]
FGF-2	rat	kidney	in vivo I/R	500 μg/kg 30 min prior to I/R, ip.	renal function ↑, apoptosis ↓,	3NT ↓, 8-OHdG ↓	[99]

^1^ H_2_O_2_ treatment to mimic I/R, ^2^ 7,8-DHF a BDNF mimetic, BDNF: brain-derived neurotrophic factor, 3NT: 3-nitrotyrosine, 4-HNE: 4-hydroxynonenal, 7,8-DHF: 7,8-dihydroxyflavone, 8-OHdG: 8-hydroxy-2-deoxyguanosine, NOS-3: endothelial nitric oxide synthase, FGF-2: Fibroblast growth factor 2, FGF-21: Fibroblast growth factor-21, FSTL-1: Follistatin-like 1, GPx: glutathione peroxidase, H/R: hypoxia/reoxygenation, IGF-1: Insulin-like growth factor 1, ip.: intraperitoneally, iv.: intravenously, LIF: leukemia inhibitory factor, MCAO: middle cerebral artery occlusion, MDA: malondialdehyde, N.A.: not applicable, NOX: NADPH oxidase, O_2_^•−^: superoxide anion, OGD: oxygen-glucose deprivation, Prdx4: Peroxiredoxin-4, SOD: superoxide dismutase, SOD-3: Extracellular superoxide dismutase [CuZn], TBARS: thiobarbituric acid reactive substances, ↑ and ↓indicate increase and decrease, respectively; ↔ indicates no changes; * indicates detection method.

## Abbreviations

BDNF	brain-derived neurotrophic factor
FGF-2	fibroblast growth factor 2
FGF-21	fibroblast growth factor-21
FSTL-1	follistatin-like
GPx	glutathione peroxidase
GSH	glutathione
HO-1	heme oxygenase-1;
IGF-1	insulin-like growth factor-1;
IL-6	interleukin-6
IL-7	interleukin-7
IL-15	interleukin-15
I/R	ischemia/reperfusion
LIF	leukemia inhibitory factor
METRLN	meteorin-like
NO	nitric oxide
NOS	NO synthase
NOS-1	neuronal NOS (nNOS)
NOS-2	inducible NOS (iNOS)
NOS-3	endothelial NOS (eNOS)
NOX	NADPH oxidase
NRF-2	nuclear factor erythroid 2–related factor 2
RNS	reactive nitrogen species
ROI	reactive oxygen intermediates
ROS	reactive oxygen species
SOD	superoxide dismutase (total)
SOD-1	cytosolic CuZnSOD
SOD-2	mitochondrial MnSOD
SOD-3	extracellular SOD
